# Engineering a genetic construct to express soluble proteins tethered to the cell membrane

**DOI:** 10.1042/BSR20260216

**Published:** 2026-09-09

**Authors:** Alejandro Ronco-Campaña, John Simms, Alan D. Goddard, Alice J. Rothnie

**Affiliations:** Aston Institute for Membrane Excellence and Department of Biosciences, Aston University, Birmingham B4 7ET, U.K.

**Keywords:** GFP, membrane tethered protein, SMALP, WALP peptide

## Abstract

Synthetic biology has significantly grown and developed in the last decades, allowing its implementation in a wide variety of fields and applications. However, there is a lack of versatile, modular tools that allow the expression of soluble proteins anchored to the cell membrane, which could be useful in a range of applications. Therefore, using a combination of computational simulations and experimental validation, we designed a genetic construct for the expression of soluble proteins tethered to the cell membrane. Using green fluorescent protein as a model soluble protein, we demonstrated that it was successfully tethered to the cell membrane and that it could be incorporated into polymer lipid nanodiscs and purified through the histidine tag. Replacement of the reporter gene with another gene of interest could lead to its application in a wide variety of fields, from creating artificial signalling or enzymatic cascades to studying lipid and protein dynamics in the cell membrane.

## Introduction

Synthetic biology represents a rapidly advancing interdisciplinary field that combines principles of biology and engineering, dedicated to the rational design and construction of novel biological systems [1]. Its versatility is rooted in the ability to reprogram living cells to perform tailored functions, enabling advances in areas such as high-added valuable metabolite production, environmental sensing, and therapeutic development [2]. By applying standardised design principles and quantitative approaches, synthetic biology provides precise control over cellular behaviour and fosters the development of innovative solutions to complex biological and industrial challenges [3]. Despite the progress achieved in synthetic biology, there still remain several limitations, including the need to develop new constructs for specific applications and poor compatibility between many already established systems. For this reason, the implementation of standardised architectures [4] and modular cloning systems [5,6] among others, are gaining relevance.

A key aspect of many synthetic biology applications is control over protein localisation. Membrane tethering offers a straightforward strategy for positioning a soluble protein at a defined cellular interface while preserving the intrinsic properties of the soluble cargo. Such an approach could be useful for allowing researchers to artificially increase local protein concentrations and precisely control biochemical reactions in both space and time. By forcing a protein into close proximity with the cell surface, it could be possible to activate specific receptor pathways, track cell membrane dynamics, and accelerate reaction rates by keeping enzymes near their intended targets. A modular membrane-tethering system could therefore provide a useful platform for studying protein behaviour in a membrane-proximal environment and for developing downstream biochemical applications.

A central requirement for this approach is a suitable membrane anchor. WALP (membrane-spanning α-helix composed of tryptophan (W), alanine (A), and leucine (L) amino acids) peptides are well-established model transmembrane (TM) helices composed of leucine and alanine residues flanked by tryptophans and have been widely used to investigate membrane insertion, helix tilt, and hydrophobic mismatch in lipid bilayers [7,8]. They therefore provide a rational starting point for the development of a minimal membrane anchor for a modular tethering construct.

In this work, we set out to develop a simple and modular protein expression system for tethering soluble proteins to the inner membrane of *Escherichia coli*. To test this concept, we designed a construct comprising a signal peptide, a TM anchor, a flexible linker, and a model soluble cargo. We then evaluated whether the resulting fusion protein could be expressed, targeted to the membrane, solubilised into polymer lipid nanodiscs, and recovered by affinity purification. In doing so, we sought to establish a proof-of-concept workflow for membrane tethering of soluble proteins that could in future be adapted to other cargos and host systems.

## Materials and methods

### WALP peptide simulations

To select the most appropriate WALP peptide for the genetic construct, molecular dynamics (MD) simulations were carried out using GROMACS [9]. The AMBER99SB-ILDN force field [10] was used to describe peptides, ions, and lipids, combined with the TIP3P water model [11]. Elongated (unfolded) WALP peptides ranging from 10 to 30 amino acids were initially positioned parallel to the membrane normal, with the centre of the peptide approximately aligned with the centre of the bilayer. Two different membrane compositions were simulated: (i) a eukaryotic-like membrane composed of 30% cholesterol, 60% 1-palmitoyl-2-oleoyl-phosphatidylcholine, and 10% 1-palmitoyl-2-oleoyl-phosphatidylserine; and (ii) a bacterial-like membrane composed of 75% POPE (1-palmitoyl-2-oleoyl-phosphatidylethanolamine), 20% POPG (1-palmitoyl-2-oleoyl-phosphatidylglycerol), and 5% cardiolipin. Initial membrane coordinates were generated using an in-house script utilizing lipid parameters based on those from Lipidbook [12], and the systems were subsequently energy minimised and underwent a production run to improve packing. Both systems were solvated with 30 water molecules per lipid and neutralised in a 150 mM NaCl buffer. Simulations were performed at 25°C and 1 bar pressure. Temperature was maintained using the V-rescale thermostat [13], and pressure was controlled using the Parrinello-Rahman barostat [14]. Long-range electrostatics were calculated using the particle mesh Ewald method [15], while van der Waals interactions were treated with a cut-off of 1.0 nm. Equations of motion were integrated with a time step of 2 fs, and bond lengths were constrained using the LINCS algorithm [16].

An initial MD simulation of 2 μs was performed for all WALP peptides. Following this, peptides that maintained at least 60% alpha-helical secondary structure—calculated using DSSP [17]—were selected for further analysis. A subsequent 1 μs simulation was performed for the passing peptides in both membrane systems. Analysis was performed on the final 500 ns of the trajectory. The final criteria for peptide selection were the maintenance of at least 80% alpha-helical character and a tilt of less than 10 degrees with respect to the membrane normal, which was calculated using an in-house script referencing the average position of the lipid phosphates. For experimental construct assembly, WALP22 was selected as a compromise anchor because it satisfied these criteria in the bacterial-like membrane while also showing favourable behaviour in the eukaryotic membrane model. The purpose of these simulations was to guide selection of the WALP TM anchor; the full OmpA–WALP–linker–His-tag–GFP fusion construct was not modelled in the present study.

### Molecular biology

For the design and study of the different genetic components, the software SnapGene Viewer and the online tool NCBI BLAST were used. The various enzymes used for DNA manipulation were purchased from New England BioLabs. The antibiotics used as a selection factor were kanamycin (kan), gentamicin, and ampicillin, at a final concentration of 50, 100, and 100 μg/ml, respectively. Glycerol stocks (20% glycerol v/v) of all the strains generated were made and stored at –80°C.

Once the genetic construct was designed, the DNA sequence was ordered from GenScript. It was obtained in a commercial plasmid, and then the membrane-tagged GFP (MTG) genetic construct was cloned into pSEVA231 (https://seva-plasmids.com), by doing a double enzymatic digestion utilizing PacI and SpeI restriction enzymes. The purification of the DNA fragments from agarose gel using the commercial kit NZYGelpure MB01102 (Nzytech). The plasmid was then transformed into the *E. coli* DH5α cloning strain using chemically competent cells from New England Biolabs and plated on Luria broth (LB)-agar plates supplemented with kan. Colonies were picked and grown in a 10 ml LB-kan overnight at 37°C and 220 rpm. Plasmids were purified from cell cultures using a commercial miniprep kit (NZYMiniprep MB01002, nzytech) following the kit protocol. The plasmids were then checked by diagnostic enzymatic digestions, and 0.7% and 1.5% agarose gels were used depending on the size of the fragment. Electrophoresis was run at 100 V for 40–60 min using an Accuris myGel™ Mini Agarose Gel Electrophoresis Apparatus. GelRed from Merck was used to stain the gels following the provider’s recommendations.

Once the plasmids were cloned in DH5α and purified, they were electroporated into the *E. coli* expression strain BL21 [18]. 80 μl of competent BL21 *E. coli* cell suspension was mixed with 100 ng of purified plasmid; the mixture was transferred to an ice-cold 0.2 cm electroporation cuvette. The cuvette containing the cell–DNA solution was incubated on ice for 5 min; after this, the cuvette was placed in the electroporator (Eppendorf Electroporator) and was pulsed at 1.8 kV. After the electric pulse, 1 ml of LB was quickly added to the cuvette, and it was incubated for 1 h at 37°C. After the incubation, the cells were plated on LB agar kan plates.

### Membrane-tagged GFP construct expression

From a glycerol stock, an LB agar-kan (100 μg/ml) plate was streaked, and a single colony was picked to inoculate a 10 ml LB-kan culture. The culture was grown at 37°C and 200 rpm shaking overnight and was used as a preinoculum to inoculate a 1 l conical flask of LB-kan. The conical flask was then incubated at 37°C, 200 rpm until it reached an optical density (OD_600_) of 0.6. The optical density was measured at 600 nm every 30 mins. At that point, 0.5 mM isopropyl β-D-1-thiogalactopyranoside (IPTG) was added to induce expression of the MTG system, and it was incubated at 25°C overnight at 200 rpm. The cells were harvested by centrifugation at 5514×***g*** for 10 min at 4°C.

### Cell disruption and membrane preparation

The cell pellets were resuspended in 10 ml purification buffer (20 mM Tris, pH 8, 150 mM NaCl) and centrifuged at 3220×***g*** for 10 min at 4°C, then resuspended in 20 ml of purification buffer supplemented with a mixture of protease inhibitors (benzamidine 1.3 μM, leupeptin 1.8 μM, and pepstatin 1 μM). The cells were then disrupted using sonication: six 30-s cycles with 20 s intervals. Debris was removed from the solution by centrifugation at 726×***g*** for 15 min at 4°C. Finally, membranes were harvested by ultracentrifugation at 100000×***g*** for 20 min at 4°C, and the pellets were resuspended in purification buffer at a concentration of 60 mg/ml (wet weight of pellet). The membrane solution was then aliquoted in 2 ml aliquots and stored at –80°C.

### Membrane solubilisation

For the membrane solubilisation 2 ml of the previously prepared *E. coli* membranes were mixed with 2 ml of 5% (w/v) SMA2000 or 2 ml of 4% (w/v) DDM in purification buffer. It was mixed for an hour with shaking at room temperature. It was then centrifuged at 100,000×***g*** and 4°C for 20 min. The supernatant was harvested. The pellet was resuspended in purification buffer supplemented with 2% (w/v) SDS, and samples were taken for further analysis. The supernatant containing solubilised membrane proteins was mixed with 400 μl bed volume of nickel-nitrilotriacetic acid (Ni-NTA) resin (HisPur, Thermo Fisher). The mix was left incubating at 4°C with agitation overnight.

### MTG purification

After the overnight incubation, the mix was poured into an empty gravity flow purification column. The initial flowthrough was recirculated into the column to ensure as much sample as possible bound to the column. Then the column was washed five times with 4 ml of purification buffer supplemented with 20 mM imidazole. Afterwards, the column was washed two times with 4 ml of purification buffer containing 40 mM imidazole. One last wash was done with 400 μl of purification buffer supplemented with 60 mM imidazole. The protein was finally eluted with 6 × 200 μl of purification buffer supplemented with 200 mM imidazole. For DDM solubilised protein, the method was the same, except all buffers were supplemented with 0.1% (w/v) DDM. The flowthrough and all the washes and elutions were kept for further analysis by SDS–PAGE and imaged for in-gel fluorescence or stained with Instant Blue (Abcam) or silver stain (Pierce) and imaged using a GelDoc Go Imaging System from BioRad. Alternatively, samples were analysed by Western blot, using either an anti-his primary antibody (R&D Systems, 1:2000) with an anti-mouse HRP secondary antibody (Cell Signalling, 1:5000) or an anti-GFP primary antibody (Cell Signalling, 1:1000) with an anti-rabbit HRP secondary antibody (Cell Signalling, 1:5000). Finally, the Western blot was developed by chemiluminescence (Pierce) and visualised using a C-DiGit scanner from Li-Cor and the Image Studio software.

## Results and discussion

### Construct design

The aim of the present study was to design a genetic expression construct for tethering soluble proteins to the cell membrane. *Escherichia coli* was selected as the host organism, as it is well characterised and widely used, simple, and cost-effective to grow [19]. The first step was to define a sequence for the TM domain. WALP peptides are a family of peptides consisting of alternating leucine and alanine amino acids flanked by tryptophan specifically designed for their insertion in lipidic bilayers [7,8]. To determine the optimal WALP-based peptide for the chimeric protein, MD simulations were performed on a series of peptides to investigate their stability and orientation within the lipid bilayer. As part of the construct design, a peptide sequence with little tilt was required. Visual inspection of the MD data revealed that hydrophobic mismatch heavily influenced the tilt angle of the TM peptide (Supplementary Table S1). It was observed that while longer peptides (WALP24–30) were accommodated well by the thicker, cholesterol-rich eukaryotic membrane model, they exhibited substantial tilting (>13°) in the thinner, bacterial-like membranes composed of POPE and POPG. This aligns with previous extensive studies on WALP peptides, which have demonstrated that when the hydrophobic length of the peptide exceeds that of the bilayer (positive hydrophobic mismatch), the helix tilts to bury its hydrophobic residues within the membrane core [7,20]. Such tilting can induce significant stress on the membrane and has been linked to the destabilisation of membrane protein complexes and potential toxicity in heterologous expression systems [21]. Conversely, peptides shorter than 16 residues failed to maintain stable α-helical folding in our simulations, likely due to an inability to span the bilayer, a phenomenon consistent with the ‘frustrated’ folding states observed in similar model peptides [22]. The simulation data showed that WALP20 gave the lowest tilt in the bacterial-like membrane model, whereas WALP24 performed best in the eukaryotic-like membrane model (Supplementary Table 1). WALP22 was selected for construct assembly as a compromise sequence because it remained within the predefined acceptance criteria in the bacterial membrane while also performing favourably in the eukaryotic membrane model, consistent with our aim of developing a design that could be adaptable beyond *E. coli*. This choice balanced immediate suitability for bacterial expression with broader prospective applicability to other membrane environments.

Next, GFP (green fluorescent protein) [23,24] was chosen as the soluble protein to be tethered to the membrane for an initial proof of concept of the expression system. The reason for this was because it enabled easy screening to determine whether the system was expressed and localised correctly, and it has been widely and successfully used in synthetic biology and in heterologous expression systems [25].

Having decided upon the TM domain and the tethered protein, there were several other key elements to decide upon for this expression construct, including the induction system, secretion signal, and purification tags. To control the protein, expression an inducible promoter was needed. There exist a wide variety of inducible promoters that have been successfully used for recombinant protein expression in *E. coli*, and from these the *E. coli* lac operon promoter (pLac) was chosen due to it being a native promoter, and its wide usage in heterologous expression regulation and synthetic biology [26]. However, it is important to acknowledge pLac basal transcription levels in the absence of inducer [27]. This leakiness is not a matter of concern in the present study due to GFP not being a toxic protein. However, for future applications where the protein may be toxic, it could lead to growth arrest or cell death before the expression induction, thus making necessary the use of a different promoter and expression control system. It has been described that an overexpression of membrane proteins, as well as proteins undergoing signalling pathways, can affect and hamper regular cell growth and functioning [28,29]. For this reason, it was decided to use a monocistronic ribosome binding site (RBS), the ST-RBS [30].

In order to target the expressed protein to the inner membrane of *E. coli*, it was decided to use the first step of the two-step secretion system present in *E. coli* [31]. Sec (general secretory pathway) and Tat (twin arginine translocation pathway) are the pathways responsible for that first step [32], and both are signalling sequence dependent [33]. As well, both, Sec and Tat pathways, have been found in bacteria, archaea, and eukaryote organism [34,35], making them an ideal choice not only for targeting the expressed protein to the inner membrane of *E. coli*, but also facilitate easier adaptation of the expression system to other organisms. The Sec system can translocate unfolded proteins to the cytoplasmic inner membrane in both co-translational and post-translational mechanisms; the latter is commonly the mechanism used by bacteria for integration of integral or TM proteins into the cytoplasmic inner membrane [36]. This situated Sec as the optimal candidate for the translocation and insertion of the MTG construct into the inner membrane. Commonly the action of a secretion-specific chaperone is needed in the Sec pathway, the chaperone SecB [37]. SecB recognises diverse peptide sequences and initiates a cascade pathway that translocates the protein to the cell membrane or to secretion. Among the SecB signalling sequences, LamB [38], MalE [39], OmpA [40,41], and OmpF [42] were considered as the signalling peptides for the MTG construct. All of them have been utilised for heterologous protein secretion [43]. It was decided to utilize OmpA as the signal peptide for the SecB pathway-mediated translocation of the MTG construct due to its wide description and usage in the literature, and previous in-house experience with it.

To allow the GFP protein to adopt the functional conformation without the interference of the WALP peptide or the membrane, a linker was added between the WALP peptide and GFP. The linker needed to be flexible so the protein could freely rotate and move to adopt and preserve its functional conformation [44]. Thus, a glycine-serine-glycine (G-S-G) linker was chosen to be used, which is an already used and characterised linker for different applications [45,46].

For purification, a histidine tag (HisTag) was selected because of its wide use and relative cheap cost [47]. Commonly the HisTags are composed of 6–10 histidines [48], but in this case it was chosen to use a 12 HisTag to make sure that it would be accessible during the protein purification process, with the knowledge that SMA2000 and related polymers decrease the affinity of His tags for nickel columns [49]. Two different locations of the HisTag within the sequence were trialled: (A) at the N-terminus of the WALP peptide and (B) at the C-terminus of the linker. It is important to note that the OmpA signalling peptide is cleaved after the translocation, if the HisTag were placed before the cleavage site it would be cleaved with the OmpA signaling peptide, making the resulting product unable to be purified, therefore for location A it was situated between the OmpA sequence and the WALP peptide. For location B it was decided to use a location that would be in the cytoplasm, but was not at the very C-terminus, because for future applications we wanted to leave the C-terminus unmodified, as often this region is important for protein-protein interactions or protein activities.

After the coding sequence, it was necessary to include a stop codon. TAA was selected as the stop codon based on *E. coli* codon usage frequency (https://www.genscript.com/tools/codon-frequency-table). For the terminator, different options were considered, including pRK34, pRK106, pRK107, and T500, among others [50], and pRK107 was selected due to previous in-house experience [51].

In order to make the construct more applicable and versatile, it was decided to flank the GFP sequence with restriction enzyme sites so the protein-coding sequence could be easily replaced with a different protein-coding sequence to generate a new TM-tagged construct. Using a type IIS enzyme [52] allowed the design of different cohesive ends on 3′ and 5′ to avoid re-ligation after enzyme digestion and increase efficiency. The required restriction sites and cohesive ends could be added to the new desired CDS by an easy PCR (polymerase chain reaction) reaction. Thus, the construct designed could have many other applications and uses and enable tethering of any chosen soluble protein to the cell membrane.

**Figure 1:**
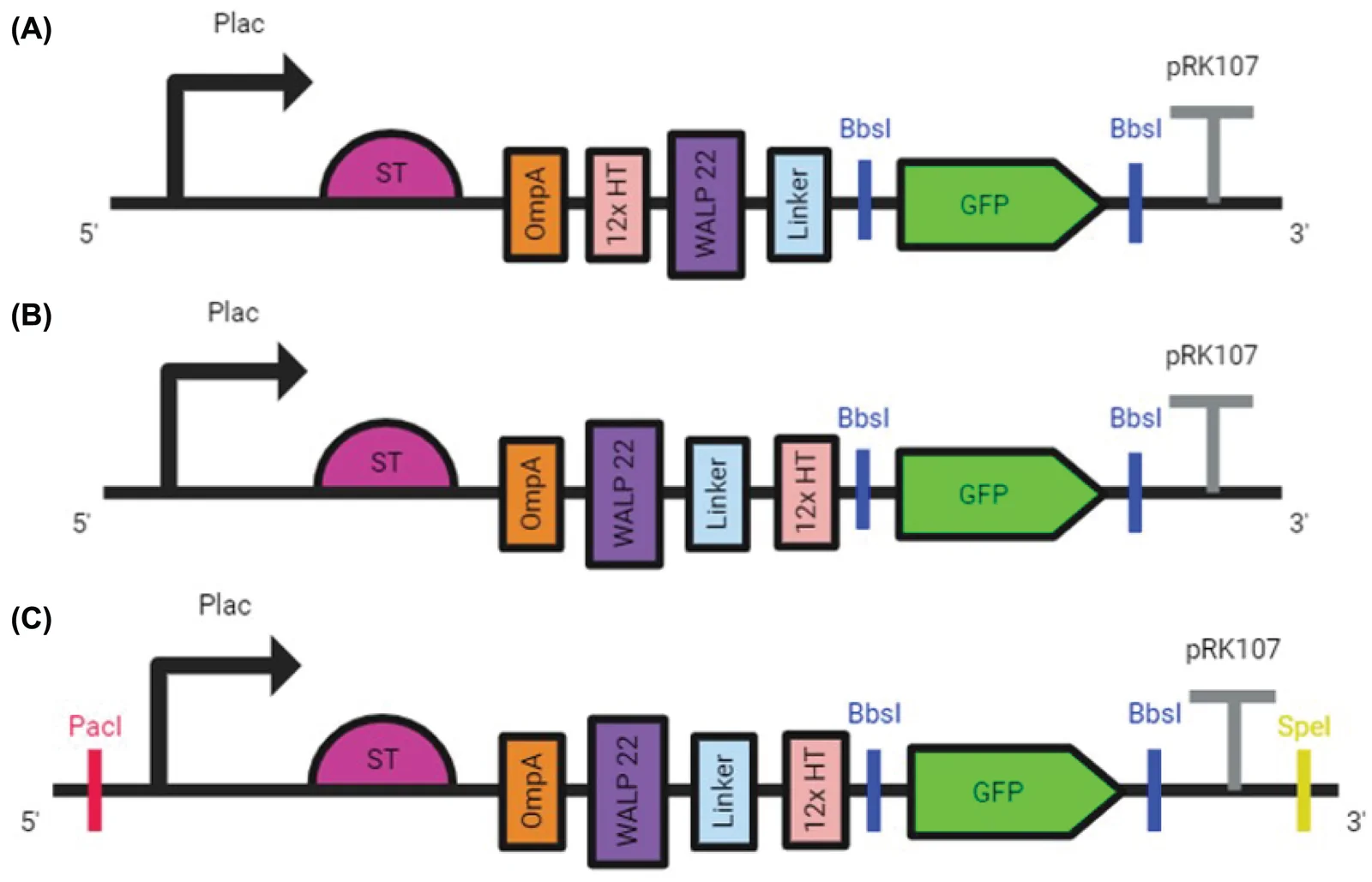
Schematic representation of the genetic construct design With Plac as the promoter, ST RBS as the RBS, OmpA signal peptide as the signalling sequence leading to the cell membrane, 12 HisTag as the purification tag, WALP22 peptide as the TM domain, G-S-G amino acid repetition as the linker, GFP as the codifying DNA sequence (CDS), and pRK107terminator as the terminator. In 5′–3′ orientation. (**A**) Presents the 12 HisTag situated between the OmpA signal peptide and the TM domain. (**B**) Presents the 12 HisTag situated between the G-S-G linker and GFP. (**C**) Schematic representation of the genetic construct design B flanked by PacI restriction site in 5′ and SpeI restriction site in 3′.

After choosing every single component, the designs of the construct are as indicated in Figure 1A,B. The present study was intentionally scoped as a proof-of-concept and therefore focused on a limited number of design permutations, namely two His-tag placements within a common architecture. Other potentially informative variables, such as alternative signal peptides, linker lengths, or TM anchor lengths, were not tested experimentally here and remain important targets for future optimisation.

**Figure 2:**
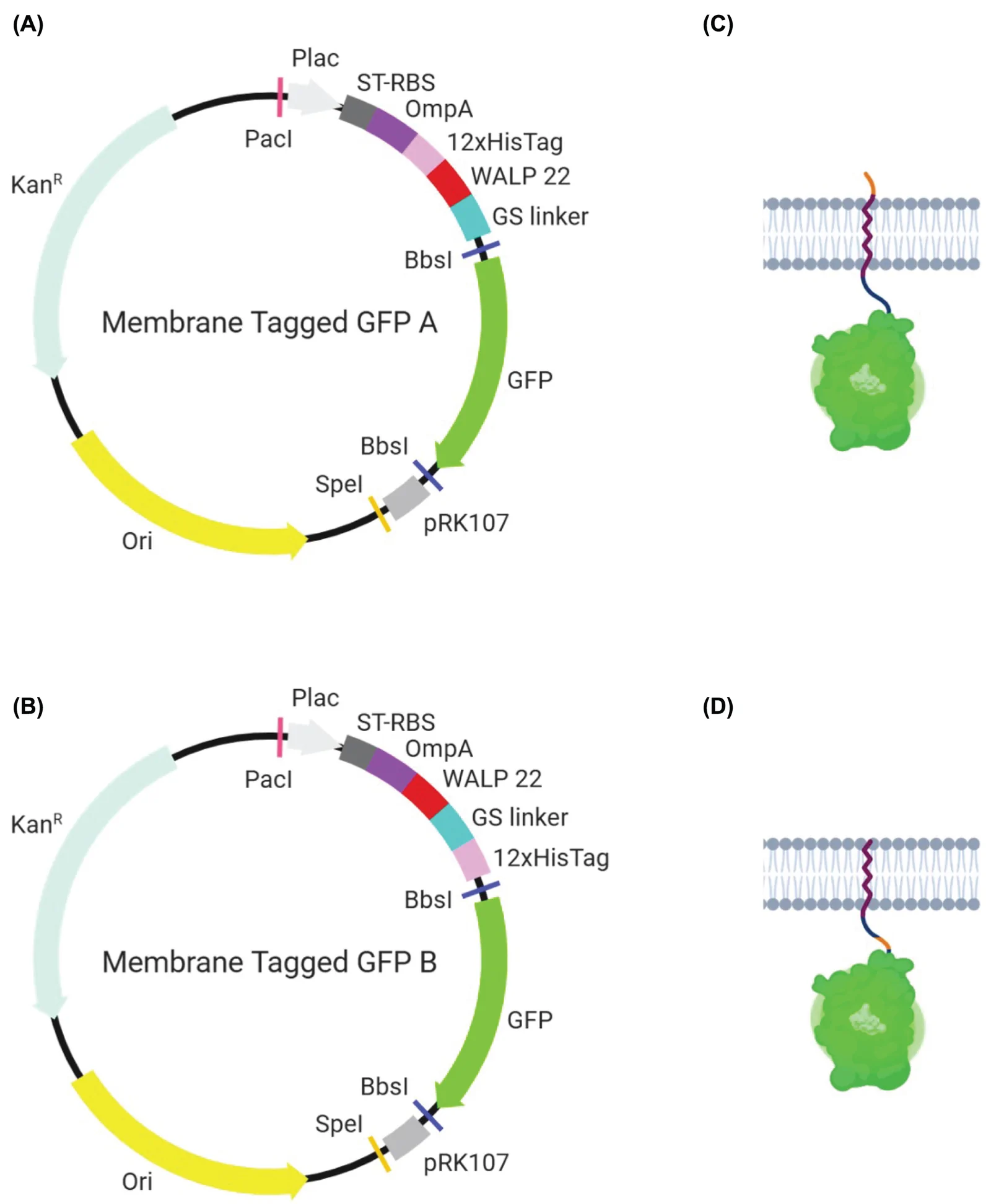
Schematic representations of the MTG constructs (**A**) Plasmid map representation of the modified pSEVA231 with the MTG A construct. Composed of replication origin pBBR1, a low copy number replication origin that is compatible with *E. coli*, and the kan resistance gene, as a selection factor. The construct includes the Plac promoter, the ST-RBS, the OmpA signal peptide, the 12 HisTag, the WALP22 peptide, the GS linker, the GFP, and the pRK107 terminator. The MTG construct is flanked by PacI (5′) and SpeI (3′) restriction sites for its compatibility with the SEVA system and the easy replacement with another cargo. The GFP coding sequence is flanked by BbsI restriction sites for its easy replacement with another CDS of interest to express tagged to the cell membrane. (**B**) Plasmid map representation of the modified pSEVA231 with the MTG B construct. (**C**) Representation of the MTG construct A expression product. HisTag in orange, cell membrane in grey, WALP22 peptide in dark red, GS linker in dark blue, and GFP in green. (**D**) Representation of the MTG construct B expression product. Cell membrane in grey, WALP22 peptide in dark red, GS linker dark blue, HisTag in orange, and GFP in green.

Once the construct was designed, it was necessary to choose the plasmid that would contain the sequence. Using the standard European vector architecture (SEVA) system for the plasmid enables a more useful and versatile expression system [4]. It would allow the antibiotic resistance cassette to be easily changed if needed; also, it would make the system easy to implement and apply in other hosts and expression systems by changing the replication origin or any other needed element, making this system adaptable to a wide range of applications and usages. To make the construct compatible with the SEVA system, it was necessary to flank the sequence with two restriction enzymes, PacI at the 5′, and SpeI at the 3′ (Figure 1C). This way the construct could easily be cloned in the cargo region of a SEVA plasmid with the desired orientation. It was decided to use the pSEVA231 as the receptor plasmid. It has kan resistance gene and the pBBR1 replication origin [53]. The pBBR1 replication origin is a low copy number replication origin, which implies a lower level of protein expression compared with a high copy number one. It already contained a BbsI enzyme restriction site (GAAGAC), which needed to be replaced (Supplementary Figure S1). The final plasmid constructs and the products they were designed to produce are shown in Figure 2.

### MTG construct expression

After the cloning of the MTG construct into pSEVA231, the plasmid was transformed into competent DH5α *E. coli* cells, and the plasmids were purified from them. The purified plasmid was transformed into the BL21 *E. coli* expression strain by electroporation.

**Figure 3:**
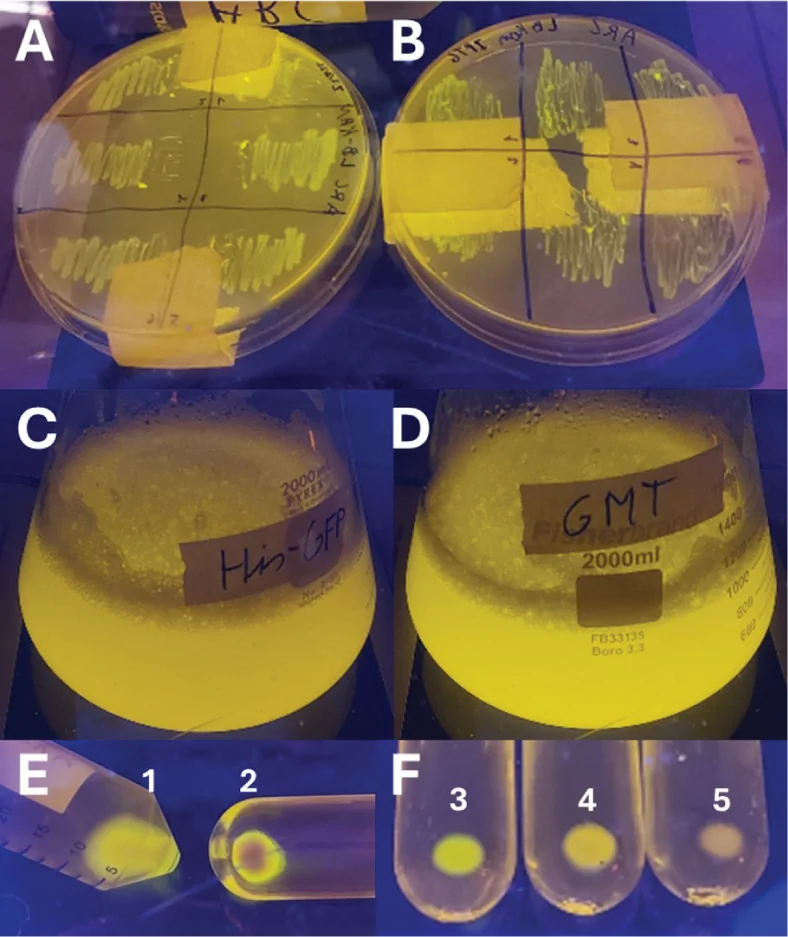
Results of MTG expression fluorescence (**A**) Clones from MTG A plated in LB agar kan plates with IPTG. (**B**) Clones from MTG B plated in LB agar kan plates with IPTG. (**C**) Overnight 1 l induced MTG A production culture. (**D**) Overnight 1 l induced MTG B production culture. (**E**) MTG B membrane prep different steps samples, (E1) cell debris collected after sonication. (E2) membrane pellet collected after final ultracentrifugation. (**F**) Membrane pellet from different strains comparison. (F3) MTG B cell membrane pellet. (F4) MTG A cell membrane pellet. (F5) cell membrane pellet from culture expressing soluble GFP. All visualised in a transilluminator.

The selected colonies were plated onto agar plates supplemented with IPTG to check the GFP construct expression (Figure 3A,B). After overnight incubation, fluorescence can be observed. Once the plasmids were checked, protein expression was carried out. A 10 ml culture of each clone was grown overnight and used as a preinoculum for 1 l cultures. When those cultures reached OD 0.6, IPTG was added to induce the protein expression, the temperature was reduced to 25°C (to try to avoid formation of inclusion bodies), and the cultures were grown overnight (Figure 3C,D).

While these images show that GFP is being produced, they do not demonstrate whether the protein is actually getting inserted into the cell membrane. A qualitatively lower biomass was observed for the MTG-expressing cultures. Although this was not quantified in the present study, this is consistent with previous reports showing that overexpression of membrane-targeted proteins can impose physiological stress in *E. coli*, including reduced growth, perturbation of membrane homeostasis, and impaired biogenesis [28,29].

To determine that the GFP was being successfully targeted to the cell membrane, a standard membrane preparation procedure was carried out. The cultures were harvested by centrifugation, disrupted by sonication, cell debris removed by low-speed centrifugation, and cell membranes isolated by ultracentrifugation. The fluorescence of the samples at each step of the membrane preparation was monitored. The cell debris following the sonication step (Figure 3E1) showed fluorescence, indicating that there is a fraction of the GFP that remains in the cytosol of the cells or that it has detached from the cell membrane during the cell disruption by sonication. However, the membrane pellet obtained in the final step of the membrane preparation (Figure 3E2) also shows fluorescence. GFP is a soluble protein that wouldn't naturally appear in the lipidic fraction that the cell membranes constitute; for this reason, the fact that GFP was present in the cell membrane pellet, could be considered as an indication of the MTG construct getting integrated into the cell membrane once expressed. When compared with the membrane pellet prepared from cells expressing regular soluble GFP, a clear difference in the fluorescence of the membrane pellet can be observed (Figure 3F), supporting that the MTG protein is getting incorporated into the membrane. We note, however, that these observations are qualitative. Direct growth analysis and fluorescence imaging of intact cells would provide important complementary evidence for the physiological impact and subcellular localisation of the construct and should be included in future optimisation of the system.

### Solubilisation and purification of MTG protein

Solubilisation of the membranes was carried out using the polymer SMA2000. This polymer has previously been demonstrated to be an effective method for extracting membrane proteins, with their surrounding lipid bilayer environment, into small nanodisc-like particles [49,54].

**Figure 4:**
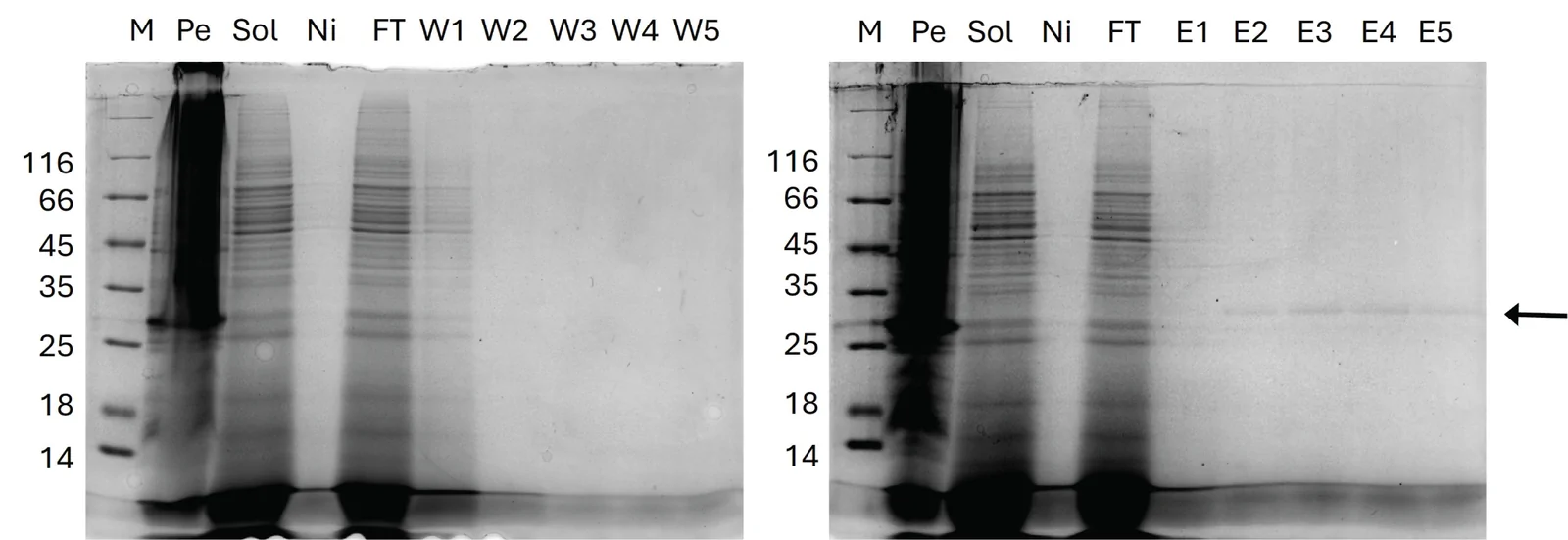
SDS–PAGE of the fractions obtained during the purification process of MTG B membrane samples M, ladder marker with sizes (kDa) indicated for each band. Pe, pellet or insoluble proteins; Sol, solubilised proteins; Ni, sample of the nickel resin used in the purification; FT, initial flow through of the purification process; W1-W5, samples of the wash steps from 1 to 5 of the purification process; E1-E5, samples of the elution steps from 1 to 5 of the purification process. Stained using InstantBlue and visualised using a BioRad GelDoc Go system. Arrow indicates the band corresponding to the purified protein.

Following solubilisation, purification of MTG was carried out using Ni-NTA affinity chromatography. The different fractions obtained during the procedure were analysed by SDS–PAGE (Figure 4).

For the MTG B construct in the elution fractions from 2 to 5, a band can be observed below the 35 kDa ladder band and around 34 kDa, which is the estimated size for the MTG protein. This showed that a protein of the correct approximate size was getting purified by using the HisTag.

**Figure 5:**
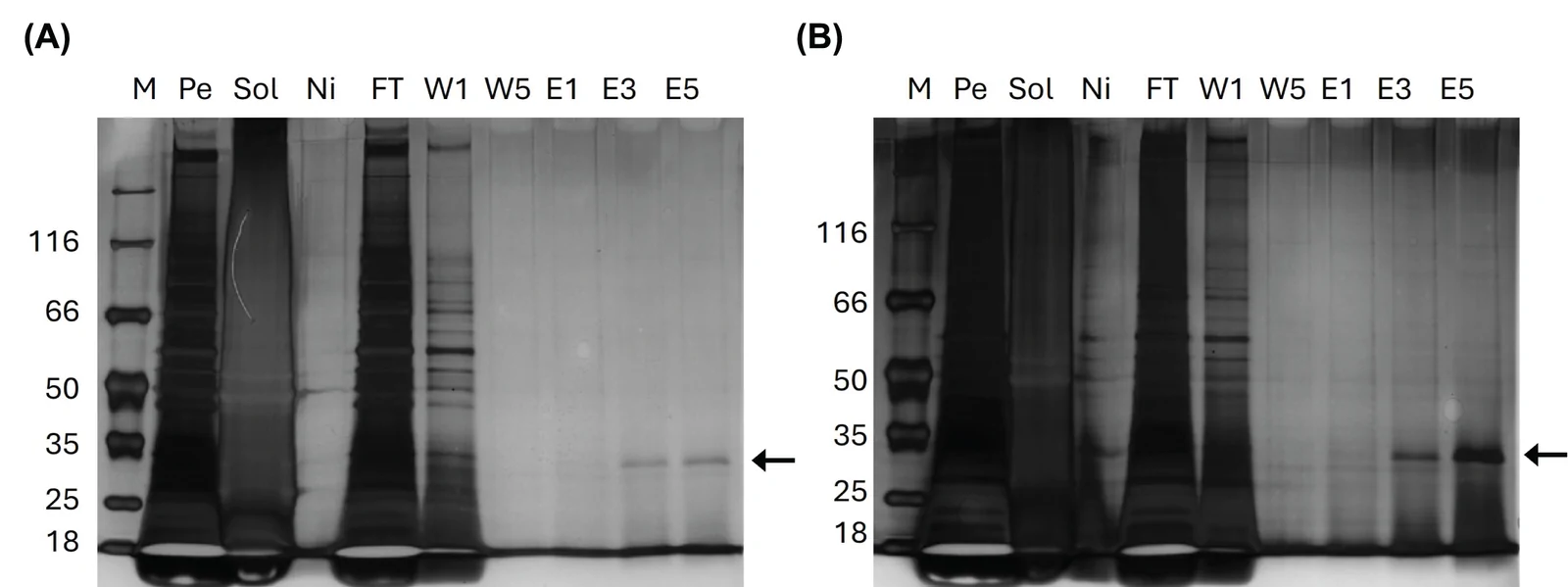
SDS–PAGE of the fractions obtained during the purification process of MTG A and MTG B membrane samples (**A**) MTG A samples. (**B**) MTG B samples. M, ladder marker with sizes indicated for each band. Pe, insoluble protein; Sol, solubilised protein; Ni, sample of the nickel resin used in the purification; FT, initial flow through of the purification process; W1 and W5, samples of the wash steps 1 and 5 of the purification process; and E1, E3, and E5, samples of the elution steps 1, 3, and 5 of the purification process. Stained using silver stain and visualised using a BioRad GelDoc Go system.

In the case of the MTG A construct, no purified protein bands were visible using InstantBlue stain. For this reason, silver staining of the gels was used, which is more sensitive (Figure 5). A band corresponding to purified MTG was now visible from both constructs, although it appeared that the MTG B (Figure 5B) gave a higher amount of purified protein than the MTG A (Figure 5A) due to the bands observed being of higher intensity. This was confirmed by running the purified samples side by side on the same gel (Supplementary Figure S2). The lower amount of protein purified for MTG A compared with MTG B might be due to the HisTag (situated at the immediate N-terminus of the WALP peptide in MTG A) interfering with the insertion of MTG A into the cell membrane, leading to a smaller quantity of it available in the membrane sample. The positioning of the HisTag at the immediate C-terminus of the linker does not interfere with MTG B insertion into the cell membrane, and despite its internal location, it is accessible for purification. An estimation of the yield of purified protein obtained would be 150-250 ug/ml. Although this is a relatively low yield, it is still within the acceptable range in the case of membrane proteins [55].

### MTG in-gel fluorescence and western blot verification

**Figure 6:**
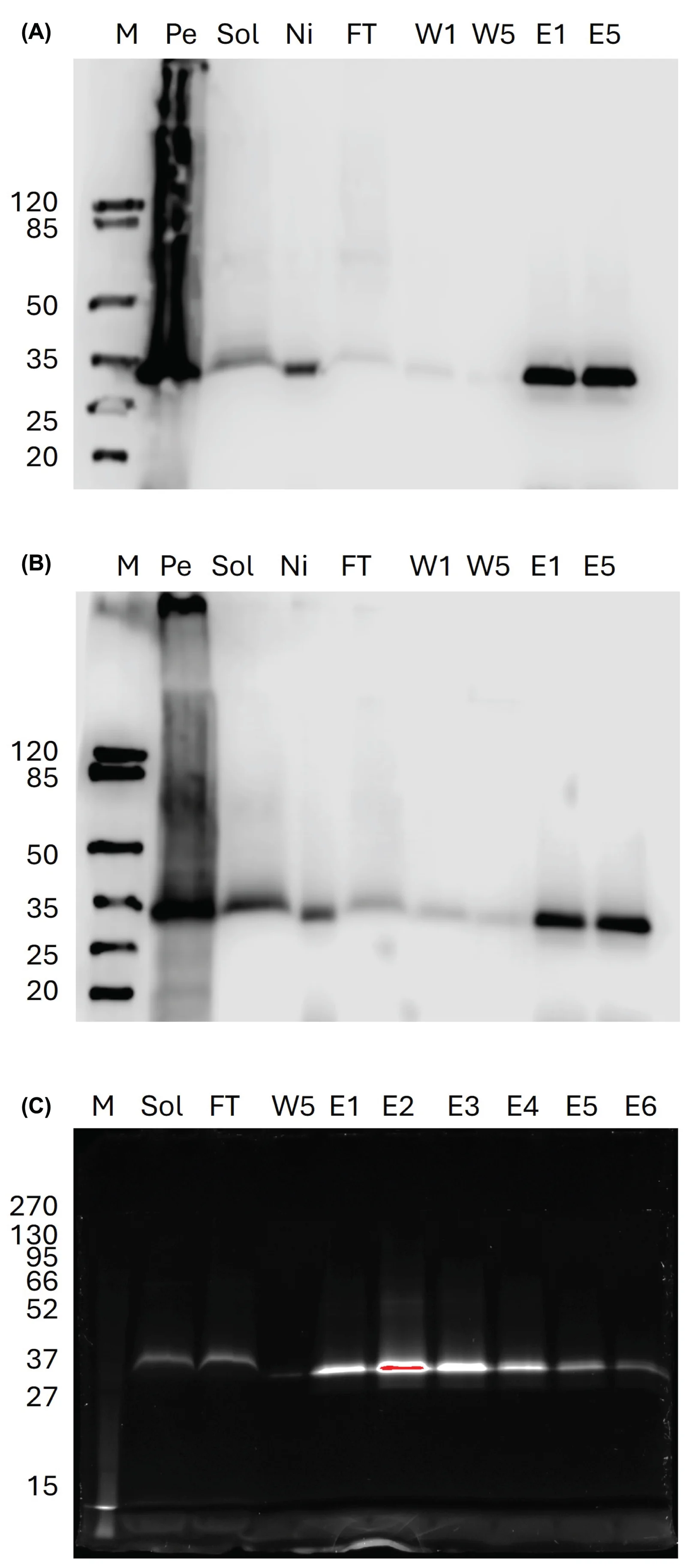
Western blot and in-gel fluorescence of the fractions obtained during purification process of MTG B (**A**) Western blot utilizing mouse anti-His antibody as the primary antibody and anti-mouse IgG as the secondary antibody. (**B**) Western blot utilizing rabbit anti-GFP as the primary antibody and anti-rabbit IgG as the secondary. (**C**) In-gel fluorescence image of purification fractions. M, ladder marker with sizes indicated for each band. Pe, insoluble protein; Sol, solubilised protein; Ni, sample of the nickel resin used in the purification; FT, initial flow through of the purification process; W1 and W5, samples of the wash steps 1 and 5 of the purification process; E1–E6, samples of the elution fractions.

**Figure 7:**
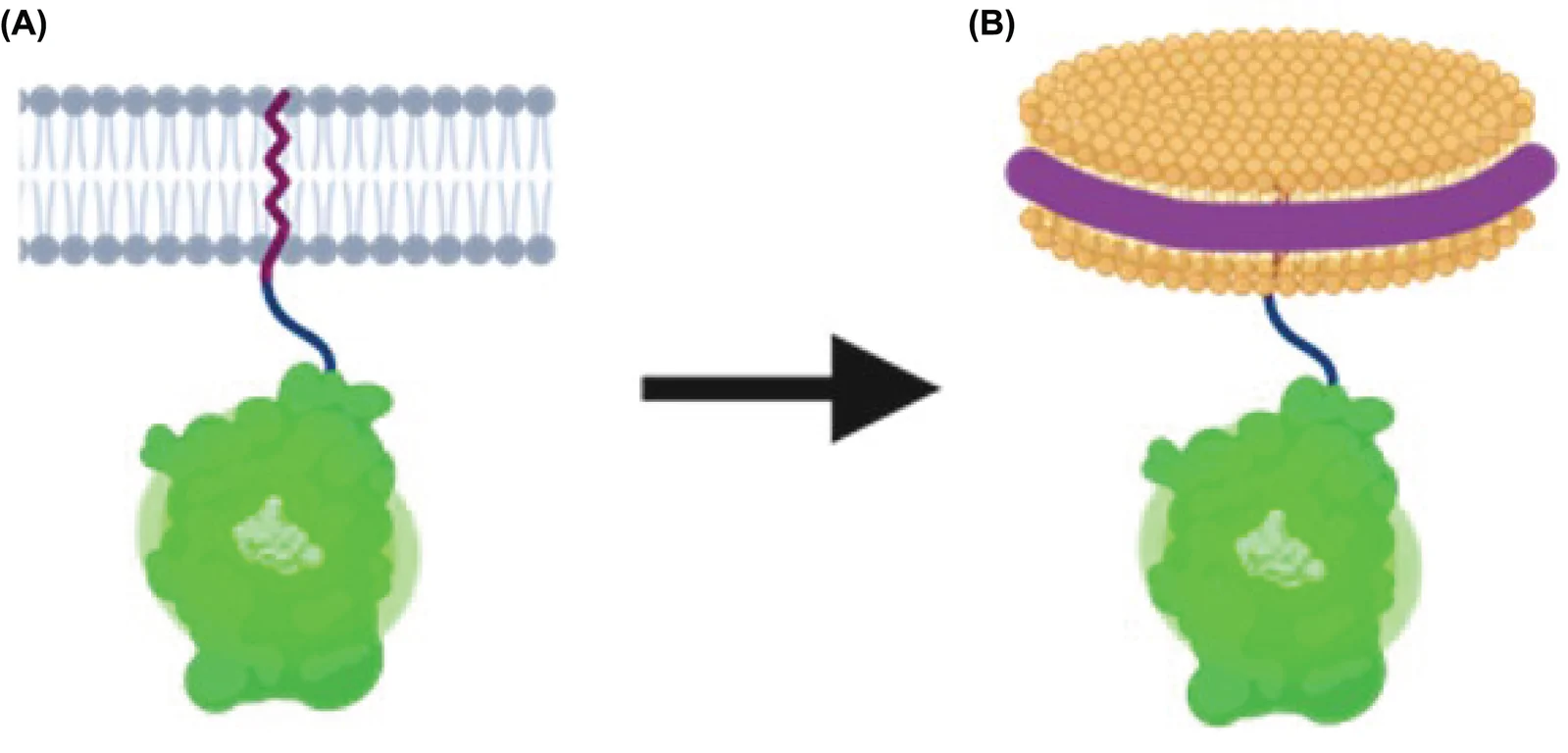
Schematic representation of the genetic construct MTG expression product (**A**) Representation of the MTG construct expression product inserted into the cell membrane. Cell membrane in grey, WALP22 peptide in dark red, GS linker and HisTag in dark blue, and GFP in green. (**B**) Representation of the MTG construct incorporated into an SMALP after membrane solubilisation utilizing SMA2000 and affinity chromatography purification.

In order to conclude the demonstration of the purified protein being indeed the MTG construct and that the GFP was correctly folded, in-gel fluorescence and Western blots of the fractions obtained during the purification process were carried out (Figure 6). This was only carried out for the MTG B samples, as it was chosen as the best design. Two different primary antibodies were utilised: mouse anti-His antibody or rabbit anti-GFP. In both cases a strong band could be seen in the elution samples matching the size of the MTG protein expression product (34 kDa) and matching the results previously observed in the SDS–PAGE gels. The fact that the Western blot was positive with both pairs of antibodies (anti-His and anti-GFP) demonstrates that the HisTag was present in the purified protein, as well as the GFP. The elution fractions from a purification were also visualised using in-gel fluorescence (Figure 6C), which showed clear fluorescent bands, showing that not only was the GFP present, but it was also correctly folded and fluorescent. In addition, the elution fractions from the purification were pooled and concentrated and the fluorescence emission spectra measured (Supplementary Figure S3). Thus, the expression and integration into the membrane of the MTG genetic construct have been demonstrated, and it can be solubilised using SMA2000, getting incorporated into SMALPs, and purified by Ni-NTA affinity chromatography targeting the HisTag (Figure 7).

Bands corresponding to the MTG construct were also observed in other fractions of the purification workflow, indicating that recovery was incomplete. Signal remaining in the pellet fraction suggests suboptimal membrane solubilisation, whereas signal in the flow-through, wash, and resin fractions indicates incomplete binding and/or elution during affinity chromatography. We therefore interpret the purification data conservatively: they demonstrate that the construct can be recovered from SMA-solubilised membranes by Ni-NTA chromatography, but they do not yet represent an optimised purification procedure. The low apparent recovery may reflect a combination of incomplete solubilisation, reduced accessibility of the His-tag within the SMALP context, and the weaker interaction of SMA-containing samples with affinity resins reported previously [49]. For comparison, the solubilisation and purification of MTG B using the conventional detergent DDM was also tested. Although it does appear to bind to the Ni-NTA resin better than the SMA2000 solubilised protein, the yield of protein is not substantially different. The in-gel fluorescence shows little difference between the two systems, but the Coomassie staining suggests a slightly higher yield with DDM. However, the degree of purity appears to be lower using DDM. (Supplementary Figure S4). Future work should therefore focus on improving both solubilisation efficiency and chromatographic recovery.

## Conclusions

In the present research, we demonstrated the successful design of a synthetic genetic construct that allows the expression of soluble proteins tethered to the cell membrane. Furthermore, it has been shown that, once expressed, the construct can be incorporated into SMALPs from the cell membrane by SMA solubilisation and purified by affinity chromatography.

The correct functioning of the genetic construct has been shown in *E. coli*, BL21 strain. *Escherichia coli* is widely used as a heterologous expression system for a myriad of different applications, ranging from laboratory research tools to industrial production of biopharmaceuticals, which enables a plethora of possible applications for the genetic construct. This would only require the replacement of the model GFP CDS by another CDS that would carry the desired function (which could be done in a single one-pot digestion-ligation reaction thanks to the restriction sites flanking it). In addition to the aforementioned applications, other possible implementations of the system could include metabolic engineering, biosynthesis and biocatalysis [19,56], protein localisation and interaction characterisation [57], biosensors development and surface display [58], artificial membrane and synthetic cell engineering [59,60], and the expression of difficult proteins, production and purification of functional proteins [61].

It should be acknowledged that the present study was limited to GFP as a model soluble cargo. GFP was selected because it provides a straightforward visual and biochemical readout for an initial proof-of-concept, but this does not guarantee equivalent behaviour for other classes of soluble proteins. In particular, enzymes, oligomeric proteins, or proteins with more complex folding requirements may differ in expression level, membrane compatibility, folding, activity, or recovery after solubilisation. Testing a broader panel of biologically relevant cargos will therefore be an important next step in evaluating the generality of the system, and optimisation would be required for any particular application. Although our main focus has been its use in *E. coli*, the genetic construct was designed to be applicable to other organisms. The Sec translocation pathway is conserved in other bacteria, archaea, and eukaryote organisms, simplifying the modifications of the genetic construct needed for its implementation on them. Likewise, the protein expression system being designed to be compatible with the SEVA system (having PacI and SpeI restriction sites flanking the construct DNA sequence) increases its versatility thanks to the variety of components available within it, such as different antibiotic resistances (for different working conditions) or different replication origins (for different host organisms), and to adjust the expression levels accordingly to possible toxicity and metabolic burden associated with it [62]. To further increase the construct flexibility and make it more customizable, it could easily be adapted to be compatible with the GoldenGate’s MoClo (modular cloning) system [5,30]. The implementation of the MoClo system would allow easy replacement of any of the elements composing the genetic construct for another, such as changing the promoter for another under a different expression control system, the RBS, CDS, signaling peptide, linker, WALP peptide, terminator, etc. If properly designed, it would also allow new elements to be added to the genetic construct. These characteristics increase the opportunity to adapt the design proposed in the present study to a myriad of biological conditions and applications.

## Supplementary Material

Supplementary Figures S1-S4 and Table S1

## Data Availability

The underlying data for the present study can be found at the Aston Explorer Data Repository doi.org/10.17036/researchdata.aston.ac.uk.00000668.

## Open Access

Open access for this article was enabled by the participation of Aston University in an all-inclusive Read & Publish agreement with Portland Press and the Biochemical Society under an agreement with JISC.

## Abbreviations

CDS: coding DNA sequence
DDM: dodecylmaltoside
GFP: green fluorescent protein
HisTag: histidine tag
IPTG: isopropyl β-D-1-thiogalactopyranoside
kan: kanamycin
LB: Luria broth
MD: molecular dynamics
MoClo: modular cloning
MTG: membrane-tagged GFP
Ni-NTA: nickel-nitrilotriacetic acid
POPE: 1-palmitoyl-2-oleoyl-phosphatidylethanolamine
POPG: 1-palmitoyl-2-oleoyl-phosphatidylglycerol
RBS: ribosome binding site
SEVA: standard European vector architecture
SMA: styrene maleic acid polymer
SMALP: SMA lipid particle
TM: transmembrane
WALP: membrane-spanning α-helix composed of tryptophan (W), alanine (A), and leucine (L) amino acids
