# Supplementary material for: Engineering a genetic construct to express soluble proteins tethered to the cell membrane
Source: Biosci Rep. 2026 Sep 9;46(9):BSR20260216. doi: 10.1042/BSR20260216 (PMC13570435; doi:10.1042/BSR20260216)
Supplement: Supplementary file 1 [file BSR-2026-0216_supp.pdf]

SUPPLEMENTARY DATA

Engineering a genetic construct to express soluble proteins tethered to the cell membrane

Alejandro Ronco-Campaña, John Simms, Alan D. Goddard & Alice J. Rothnie*

Aston Institute for Membrane Excellence and Department of Biosciences, Aston University, Birmingham, B4 7ET, UK.

Supplementary Table 1

Supplementary Table 1. Simulated tilt angles of WALP peptides in bacterial and eukaryotic membrane models. *The table displays the average tilt angle (degrees) relative to the membrane normal calculated over the final 500 ns of a 1 µs molecular dynamics simulation. Peptide Name/Length, total amino acid count of the WALP variant (WWA-[LA]_n-WW). Status, outcome of the initial 2µs folding screen; peptides with lengths 10–15 and 27–30 are labelled as "Failed Folding" due to maintaining <60% alpha-helical structure and were excluded from further analysis (N/A). Bacterial Tilt, average angle in a membrane composed of 75% POPE, 20% POPG, and 5% Cardiolipin. Mammalian Tilt, average angle in a membrane composed of 30% Cholesterol, 60% POPC, and 10% POPS. Optimal candidates (WALP20 for bacterial; WALP24 for eukaryotic) were identified based on a tilt <10° and maintenance of >80% helical character.*

**Peptide Name**	**Length (AA)**	**Status**	**Bacterial Tilt (deg)**	**Mammalian Tilt (deg)**
**WALP10**	10	**Failed Folding**	N/A	N/A
**WALP11**	11	**Failed Folding**	N/A	N/A
**WALP12**	12	**Failed Folding**	N/A	N/A
**WALP13**	13	**Failed Folding**	N/A	N/A
**WALP14**	14	**Failed Folding**	N/A	N/A
**WALP15**	15	**Failed Folding**	N/A	N/A
**WALP16**	16	Simulated	4.1	2.5
**WALP17**	17	Simulated	3.8	2.3
**WALP18**	18	Simulated	3.2	2.1
**WALP19**	19	Simulated	2.9	1.9
**WALP20**	**20**	**Simulated (Best Bacterial – good overall)**	**1.8**	**1.8**
**WALP21**	21	Simulated	4.5	1.7
**WALP22**	22	Simulated	7.2	1.6
**WALP23**	23	Simulated	9.8	1.5
**WALP24**	**24**	**Simulated (Best Mammalian)**	13.5	**1.2**
**WALP25**	25	Simulated	16.8	3.4
**WALP26**	26	Simulated	19.2	5.7
**WALP27**	27	**Failed Folding**	N/A	N/A
**WALP28**	28	**Failed Folding**	N/A	N/A
**WALP29**	29	**Failed Folding**	N/A	N/A
**WALP30**	30	**Failed Folding**	N/A	N/A

Supplementary Figure 1

In order to be able to use the BbsI enzyme to replace the CDS it was necessary to remove the restriction site that existed within the plasmid sequence.

The coding sequence in frame is caGAAGACa coding for Gln-Lys-Thr. The codons coding for Gln are CAG and CAA; for Lys are AAG and AAA; and for Thr are ACG, ACA, ACC, and ACT. It was decided to change the codon for Lys from AAG to AAA to remove the BbsI enzyme restriction site. That way the sequence remains in frame and codes for the same amino acids. This change was chosen because it's in the centric position of the sequence that needed modification and it's an exchange between two purines. Other considered changes were from CAG to CAA for Gln, and ACA to ACG for Thr. It was also evaluated the option of making more than one nucleotide modification, but it was decided to maintain the amount changed as low as possible in order to having the least impact possible in the functionality of the sequence and the plasmid itself.

A

```
ctaccggcgcggcagcgttacccgtgtcggcggctccaacggctcgccatcgtccagaaaac
acggctcatcgggcatcggcaggcgctgctgcccgcgccgttcccattcctccgtttcggtc
aaggctggcaggtctggttccatgcccggaatgccgggctggctgggcggctcctcgccggg
gccggtcggtagttgctgctcgcccggatacagggtcgggatgcggcgcaggtcgccatgcc
ccaacagcgattcgtcctggtcgtcgtgatcaaccaccacggcggcactgaacaccgacagg
cgcaactggtcgcggggctggccccacgccacgcggtcattgaccacgtaggccgacacggt
gccggggccgttgagcttcacgacggagatccagcgctcggccaccaagtccttgactgcgt
attggaccgtccgcaaagaacgtccgatgagcttggaaagtgtcttctggctgaccaccacg
gcgttctggtggcccatctgcgccacgaggtgatgcagcagcattgccgccgtgggtttcct
cgcaataagcccggcccacgcctcatgcgctttgcgttccgtttgcacccagtgaccgggct
tgttcttggcttgaatgccgatttctctggactgcgtggccat
```

B

```
ctaccggcgcggcagcgttacccgtgtcggcggctccaacggctcgccatcgtccagaaaac
acggctcatcgggcatcggcaggcgctgctgcccgcgccgttcccattcctccgtttcggtc
aaggctggcaggtctggttccatgcccggaatgccgggctggctgggcggctcctcgccggg
gccggtcggtagttgctgctcgcccggatacagggtcgggatgcggcgcaggtcgccatgcc
ccaacagcgattcgtcctggtcgtcgtgatcaaccaccacggcggcactgaacaccgacagg
cgcaactggtcgcggggctggccccacgccacgcggtcattgaccacgtaggccgacacggt
gccggggccgttgagcttcacgacggagatccagcgctcggccaccaagtccttgactgcgt
attggaccgtccgcaaagaacgtccgatgagcttggaaagtgtTttctggctgaccaccacg
gcgttctggtggcccatctgcgccacgaggtgatgcagcagcattgccgccgtgggtttcct
cgcaataagcccggcccacgcctcatgcgctttgcgttccgtttgcacccagtgaccgggct
tgttcttggcttgaatgccgatttctctggactgcgtggccat
```

Supplementary Figure 1. Elimination of BbsI restriction site in the pBBR1 replication origin DNA sequence by changing a nucleotide. *(A) Original pBBR1 replication origin DNA sequence, BbsI restriction site in yellow.* ***(B)*** *Modified pBBR1 replication origin DNA sequence, BbsI restriction site in yellow, nucleotide changed in green.*

Supplementary Figure 2

To verify the relative yield of purified MTG samples from each construct they were run alongside each other on the same gel. It can be seen that the amount of protein in the elutions of MTG B is indeed higher, by approximately 2-fold.

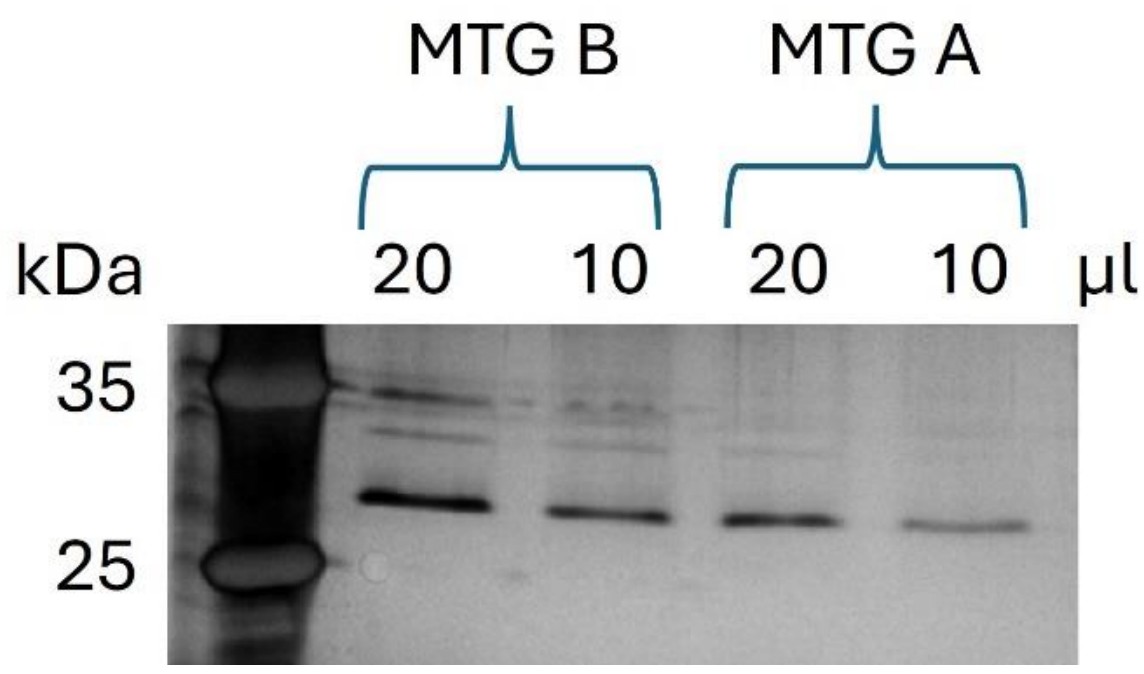

Supplementary Figure 2. SDS-PAGE comparing the elution fractions obtained during purification process of MTG A and MTG B membrane samples. *Stained using silver stain and visualised using a BioRad GelDoc Go system.*

Supplementary Figure 3

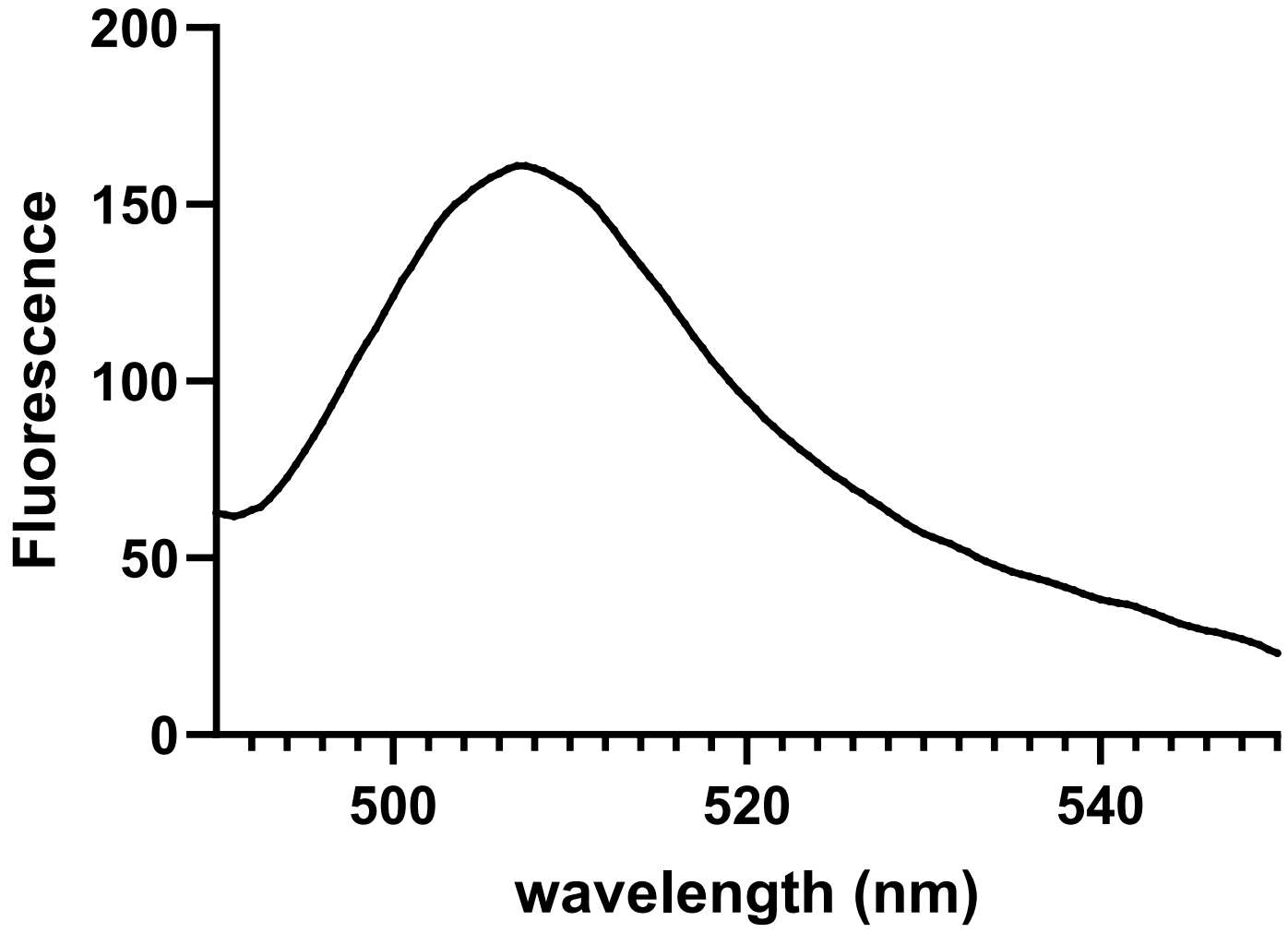

Supplementary Figure 3. Fluorescence spectra of purified MTG. *Elution fractions from SMALP purified MTG B were pooled and concentrated to 100µl using a centrifugal filter concentrator. Fluorescence spectra obtained using a Perkin Elmer LS55 fluorimeter. Excitation 488±10nm, emission 490-550±10nm.*

Supplementary Figure 4

For comparison with the SMA2000 purification of MTG, a solubilisation and purification with the conventional detergent DDM was carried out.

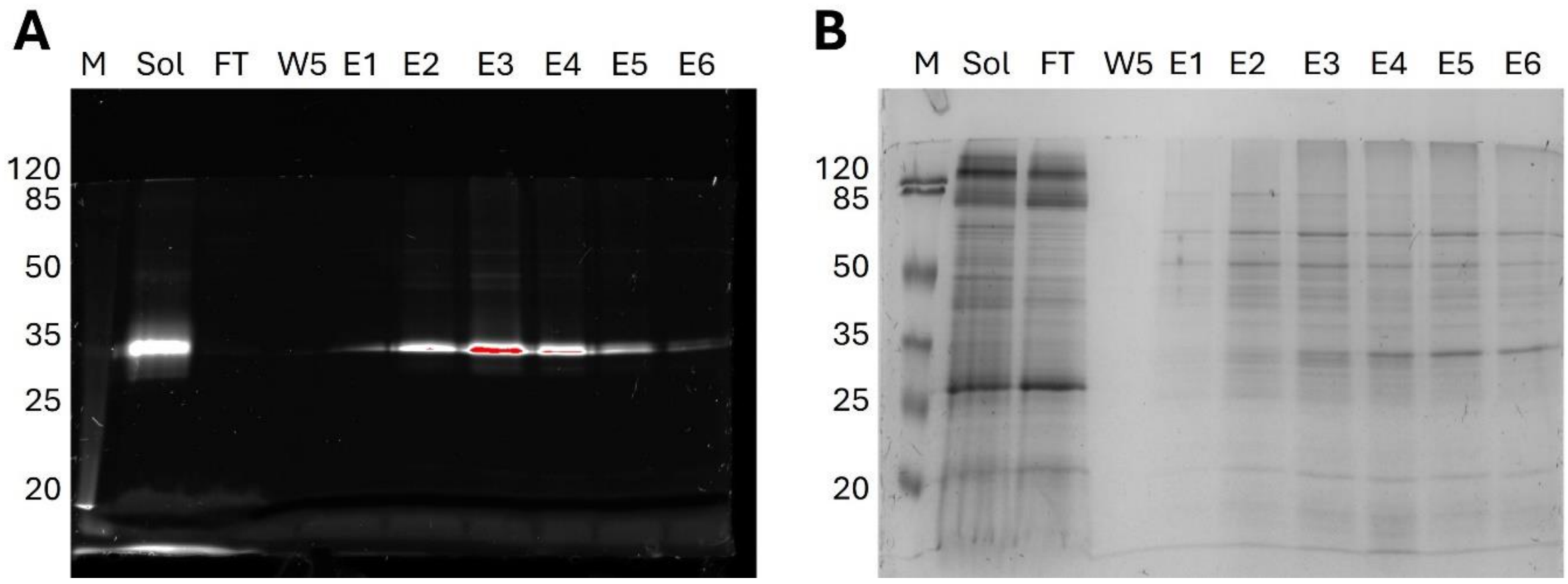

Supplementary Figure 4. SDS-PAGE of the fractions obtained during purification of MTG B using DDM. *M, ladder marker with sizes(kDa) indicated for each band. Sol, solubilised proteins; FT, initial flow through of the purification process; W5, sample of the 5th wash step; E1-E6, samples of the elution fractions 1 to 6.* ***(A)*** *Imaged using in-gel fluorescence.* ***(B)*** *Stained using InstantBlue.*

Supplementary Information 1

Full DNA sequence of the MTG expression cassette (1076 bp):

TTAATTAATTTACACTTTATGCTTCCGGCTCGTATGTTGTGACCTAAGGAGGTAAATAATGA
AAAAGACAGCTATCGCGATTGCAGTGGCACTGGCTGGTTTCGCTACCGTAGCGCAGGCCTGG
TGGGCGCTGGCGCTGGCGCTGGCCCTGGCACTGGCACTGGCTCTGGCACTGTGGTGGGGCAG
CGGCAGCGGCAGCGGCAGCGGCAGCGGCAGCCACCACCACCACCACCACCACCACCACCACC
ACCACGAAGACAATTACCGTAAAGGAGAAGAACTTTTCACTGGAGTTGTCCCAATTCTTGTT
GAATTAGATGGTGATGTTAATGGGCACAAATTTTCTGTCAGTGGAGAGGGTGAAGGTGATGC
AACATACGGAAAACTTACCCTTAAATTTATTTGCACTACTGGAAAACTACCTGTTCCATGGC
CAACACTTGTCACTACTTTCGGTTATGGTGTTCAATGCTTTGCGAGATACCCAGATCATATG
AAACAGCATGACTTTTTCAAGAGTGCCATGCCCGAAGGTTATGTACAGGAAAGAACTATATT
TTTCAAAGATGACGGGAACTACAAGACACGTGCTGAAGTCAAGTTTGAAGGTGATACCCTTG
TTAATAGAATCGAGTTAAAAGGTATTGATTTTAAAGAAGATGGAAACATTCTTGGACACAAA
TTGGAATACAACTATAACTCACACAATGTATACATCATGGCAGACAAACAAAAGAATGGAAT
CAAAGTTAACTTCAAAATTAGACACAACATTGAAGATGGAAGCGTTCAACTAGCAGACCATT
ATCAACAAAATACTCCAATTGGCGATGGCCCTGTCCTTTTACCAGACAACCATTACCTGTCC
ACACAATCTGCCCTTTCGAAAGATCCCAACGAAAAGAGAGATCACATGGTCCTTCTTGAGTT
TGTAACAGCTGCTGGGATTACACATGGCATGGATGAACTATACAAATAACAGATTGTCTTCG
TAATCGTTAATCCGCAAATAACGTAAAAACCCGCTTCGGCGGGTTTTTTTATGGGGGGAGTT
TAGGGAAAGAGCATTTGTCAACTAG

PacI: TTAATTAA

Plac: TTTACACTTTATGCTTCCGGCTCGTATGTTG

ST-RBS: TGACCTAAGGAGGTAAATA

Start codon: ATG

OmpA signal peptide:
AAAAAGACAGCTATCGCGATTGCAGTGGCACTGGCTGGTTTCGCTACCGTAGCGCAGGCC

WALP22 peptide:
TGGTGGGCGCTGGCGCTGGCGCTGGCCCTGGCACTGGCACTGGCTCTGGCACTGTGGTGG

GS linker: GGCAGCGGCAGCGGCAGCGGCAGCGGCAGCGGCAGC

12xHisTag: CACCACCACCACCACCACCACCACCACCACCACCAC

BbsI recognition site: GAAGAC

BbsI restriction site 1: TTAC

GFP:
AAAGGAGAAGAACTTTTCACTGGAGTTGTCCCAATTCTTGTTGAATTAGATGGTGATGTTAA
TGGGCACAAATTTTCTGTCAGTGGAGAGGGTGAAGGTGATGCAACATACGGAAAACTTACCC
TTAAATTTATTTGCACTACTGGAAAACTACCTGTTCCATGGCCAACACTTGTCACTACTTTC
GGTTATGGTGTTCAATGCTTTGCGAGATACCCAGATCATATGAAACAGCATGACTTTTTCAA

GAGTGCCATGCCCGAAGGTTATGTACAGGAAAGAACTATATTTTTCAAAGATGACGGGAACT
ACAAGACACGTGCTGAAGTCAAGTTTGAAGGTGATACCCTTGTTAATAGAATCGAGTTAAAA
GGTATTGATTTTAAAGAAGATGGAAACATTCTTGGACACAAATTGGAATACAACTATAACTC
ACACAATGTATACATCATGGCAGACAAACAAAAGAATGGAATCAAAGTTAACTTCAAAATTA
GACACAACATTGAAGATGGAAGCGTTCAACTAGCAGACCATTATCAACAAAATACTCCAATT
GGCGATGGCCCTGTCCTTTTACCAGACAACCATTACCTGTCCACACAATCTGCCCTTTCGAA
AGATCCCAACGAAAAGAGAGATCACATGGTCCTTCTTGAGTTTGTAACAGCTGCTGGGATTA
CACATGGCATGGATGAACTATACAAA

STOP codon: TAA

BbsI restriction site 2: CAGA

BbsI recognition site: GTCTTC

pRK107 terminator:
GTAATCGTTAATCCGCAAATAACGTAAAAACCCGCTTCGGCGGGTTTTTTTATGGGGGGAGT
TTAGGGAAAGAGCATTTGTCA

SpeI: ACTAGT

SUPPLEMENTARY DATA

Engineering a genetic construct to express soluble proteins tethered to the cell membrane

Alejandro Ronco-Campaña, John Simms, Alan D. Goddard & Alice J. Rothnie*

Aston Institute for Membrane Excellence and Department of Biosciences, Aston University, Birmingham, B4 7ET, UK.

Uncropped Western blots

Anti-his

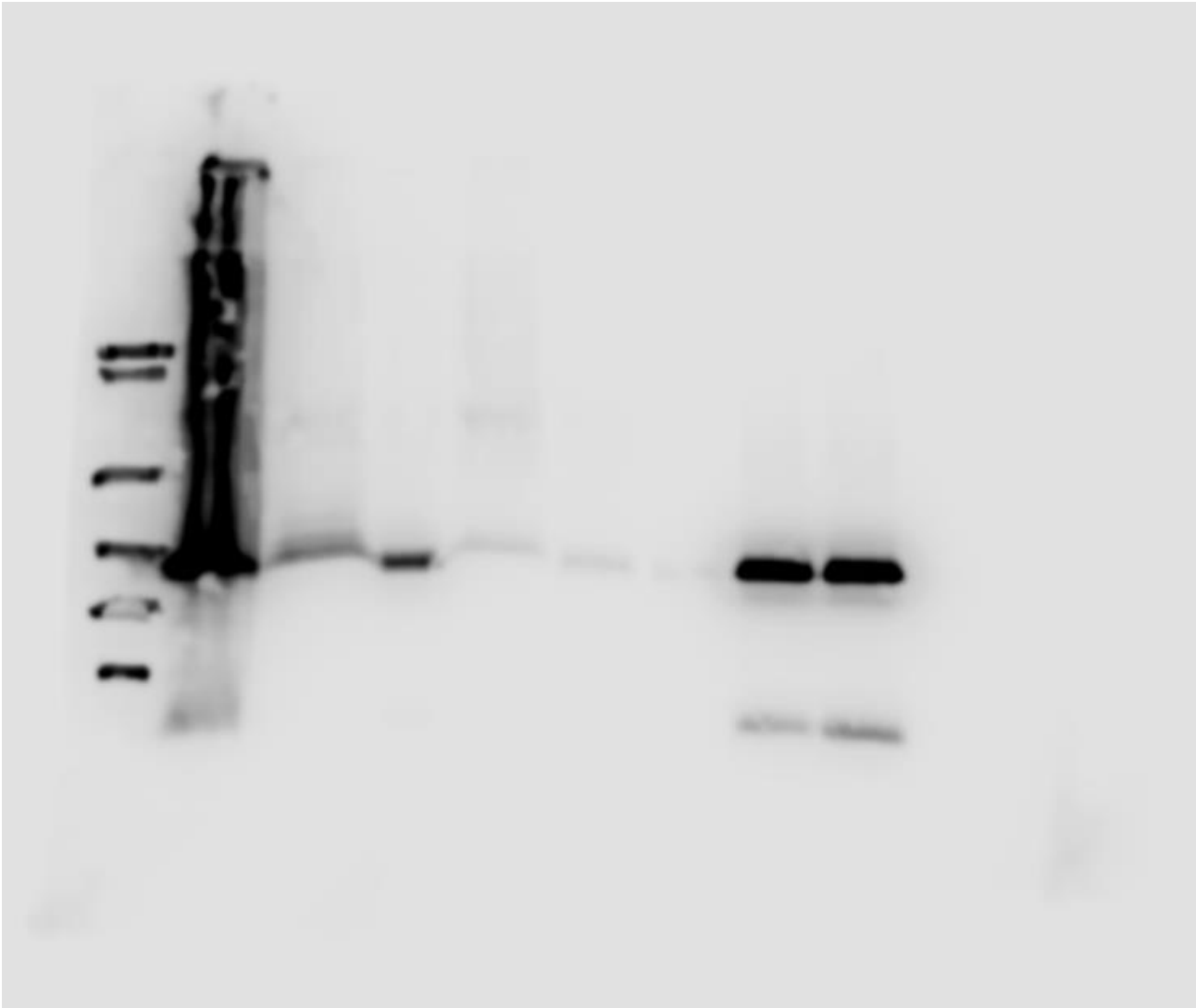

Anti-GFP

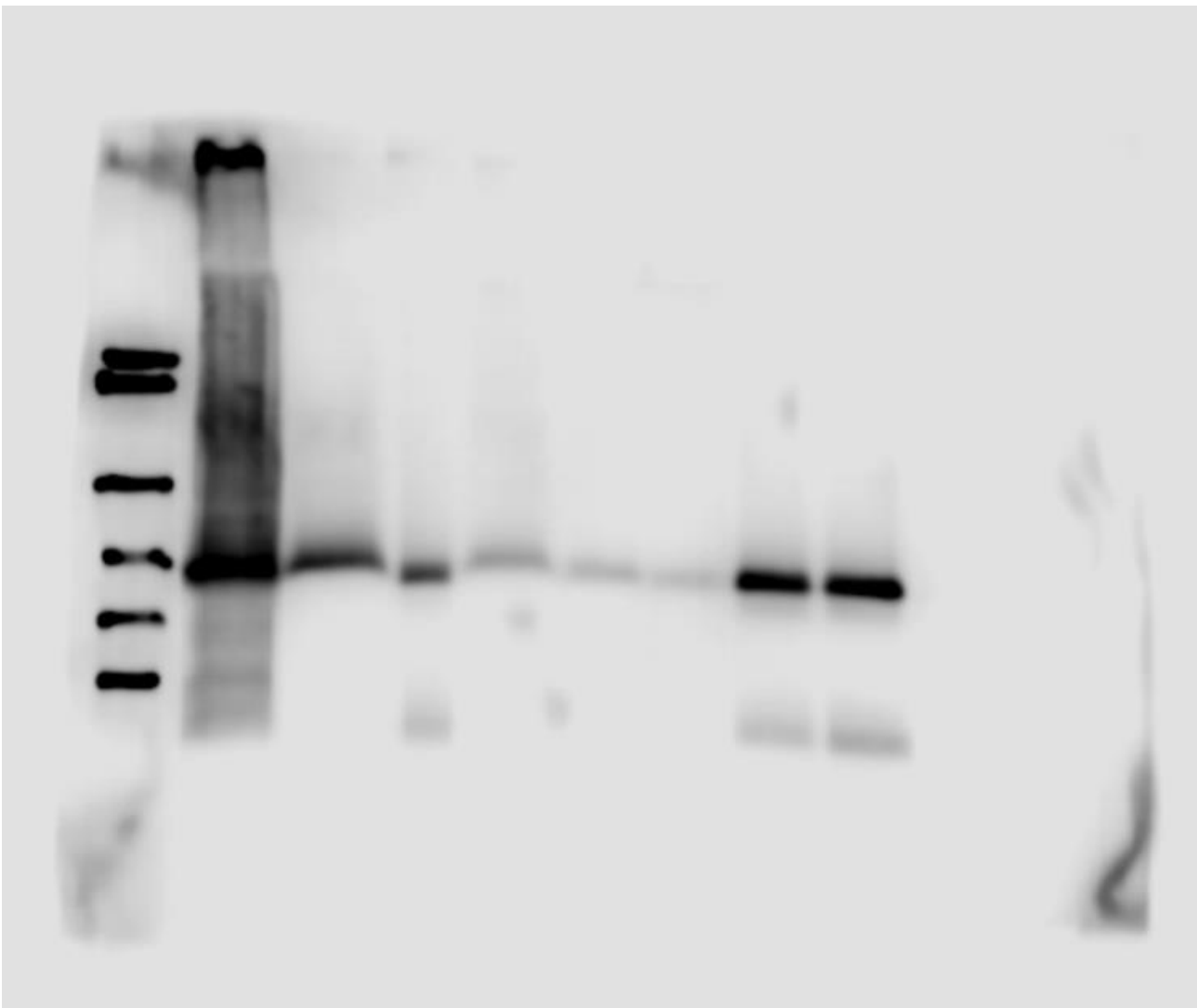

Uncropped in-gel fluorescence

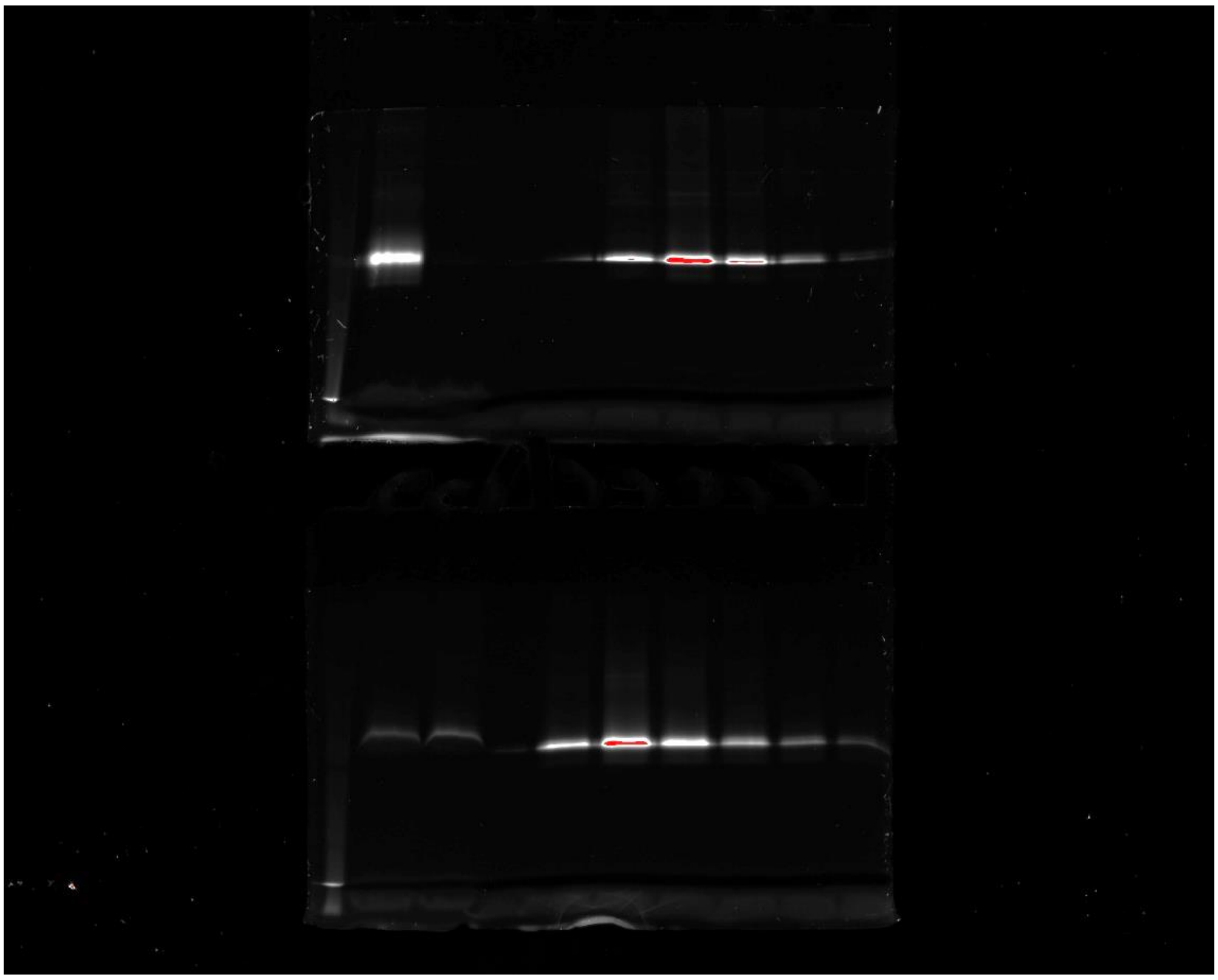

DDM

SMA2000
